# Insertion of Platinum Nanoparticles into MoS_2_ Nanoflakes for Enhanced Hydrogen Evolution Reaction

**DOI:** 10.3390/ma11091520

**Published:** 2018-08-24

**Authors:** Dan Li, Yang Li, Bowei Zhang, Yu Hui Lui, Sivaprasad Mooni, Rongsheng Chen, Shan Hu, Hongwei Ni

**Affiliations:** 1 The State Key Laboratory of Refractories and Metallurgy, Key Laboratory for Ferrous Metallurgy and Resources Utilization of Ministry of Education, Wuhan University of Science and Technology, Wuhan 430081, China; danliwust@163.com (D.L.); liyang2468@wust.edu.cn (Y.L.); sivaphdchem@gmail.com (S.M.); 2 Department of Mechanical Engineering, Iowa State University, Ames, IA 50011, USA; boweiz@iastate.edu (B.Z.); luiyuhui1992@gmail.com (Y.H.L.); 3 Electroanalytical Lab., Department of Chemistry, Sri Venkateswara University, Tirupati 517502, India; 4 School of Chemical Engineering and Technology, Wuhan University of Science and Technology, Wuhan 430081, China; chenrs@126.com

**Keywords:** MoS_2_, HER, Pt counter electrode, insertion, stainless steel mesh

## Abstract

Pt as a chemical inert metal has been widely applied as the counter electrode in various electrochemical measurements. However, it can also be dissolved and redeposit to the working electrode under certain electrochemical circumstances. Herein we demonstrated a cyclic voltammetry (CV) cycling method to synthesize a catalyst comprising inserted Pt nanoparticles into MoS_2_ nanoflake stack structures on stainless steel mesh (SSM). The binder-free composite structure exhibits significantly enhanced hydrogen evolution reaction (HER) catalytic activity with an overpotentials of 87 mV at 10 mA cm^−2^. The deposited Pt nanoparticles significantly enhance the catalytic activity through changing the structure of MoS_2_ and increasing the amount of active sites. This work provides a new way forward for rational design of the nano-electrocatalysts.

## 1. Introduction

To address the environmental crisis of global warming and energy shortage, all kinds of renewable energies such as wind, water and solar energy have been regarded as promising substitutes for fossil energy [1,2,3,4]. Hydrogen gas (H_2_), with zero carbon emission and a high combustion value, has been considered to be a green and high energy source as it can be produced by electro-splitting of water, which can convert electric energy into chemical energy for easier storage and delivery [5]. Up to now, noble-metal platinum (Pt) based electrocatalysts remain as the benchmark for hydrogen evolution reaction (HER) owing to the low overpotential and small Tafel slope. Unfortunately, its widespread utilization have been greatly restricted by the skyrocketing prices. Therefore, to develop noble-metal-free HER catalysts with high catalytic efficiency, long-term durability and low cost, many metal and metallic compounds with various nanostructures have been exploited [6]. Transition metal composites [7,8], transition metal carbide [9,10], phosphides [11,12], and chalcogenides [13,14,15,16] have received extensive research interest.

Over the past decade, molybdenum disulfide (MoS_2_) has attracted a great amount of attention because of its extraordinary ability for accepting electrons and protons [13,15]. This material exhibits good corrosion resistance compared with other most Transition metal composites in acid solution and similar electronic properties with Pt-group metals [16,17]. What’s more, the hydrogen binding energy of MoS_2_ was calculated to be similar to that of Pt [18,19] and the reaction activity of MoS_2_ can be tuned by the density of exposed edge sites. The inert basal plane with limited amount of edge active sites greatly limits its catalytic activity. In recent years, some approaches such as chemical interlayer intercalation [14,20], 2H-to-1T phase conversion [15,21] and large surface structural design [22,23] have been reported as effective methods to reduce the HER overpotentials and Tafel slope of MoS_2_.

Pt metal has been prevalently used as a counter electrode in various electrochemical measurements due to its chemical inertness. However, Pt can also be oxidized and dissolved under certain chemical or electrochemical circumstances. The dissolution of Pt is most significant when highly oxidative potential is applied to the Pt counter electrode, while repetitive oxidation and reduction cycles further aggravate the dissolution of Pt [24,25]. Pt dissolution and redeposition are easier in acidic media than that in alkaline medium during electrochemical process [26]. In HER process, cyclic voltammetry (CV) scans are inevitably applied to pretreat the surface of electrocatalysts and stabilize the polarization curve. Therefore, without ion exchange membrane to separate the working electrode from the counter electrode, Pt counter electrode would be unavoidably dissolved and redeposited onto the working electrode and would affect the HER catalytic activity of the working electrode.

Herein, we demonstrate a facile and cost-effective route to grow MoS_2_ nanoflakes directly on the stainless steel mesh (SSM) substrates via a hydrothermal process. SSM is a widely used engineering product with a high physical durability and chemical resistance in both basic and acidic environments [27,28]. Additionally, it has a relatively low electrical resistivity of around 70 mΩ cm and the elements of Mo, Ni, Cr are also in favor of the HER activity. Then Pt plate was used as counter electrode to introduce Pt nanoparticles into MoS_2_ nanoflakes by multiple CV cycles in 0.5 mol L^−1^ H_2_SO_4_ aqueous solution. The resultant composite electrode is denoted as Pt-MoS_2_/SSM. The composite structure exhibits significantly enhanced intrinsic catalytic activity toward HER, compared with MoS_2_/SSM and Pt/SSM. The enhanced HER performance of Pt-MoS_2_/SSM is ascribed to the interaction of Pt and MoS_2_.

## 2. Materials and Methods

### 2.1. Materials and Chemicals

316L SSM (1 cm × 2.5 cm, 200 mesh) was purchased from good fellow (Cambridge, Ltd., Shanghai, China). Pt plate (0.6 cm × 0.2 cm), Na_2_MoO_4_·2H_2_O (99%), CH_4_N_2_S (99%), H_2_SO_4_ (95%), HCL (37%), ethanol were purchased from Sinopharm Chemical Reagent Co., Ltd., Shanghai, China. All chemicals were analytical grade and used as received without further purification. The deionized water (~18.2 mΩ.cm) used throughout all experiments was purified by Milli-Q system (Thermo Fisher Scientific, Massachusetts, MA, USA).

### 2.2. Synthesis of Pt-MoS_2_/SSM, MoS_2_/SSM and Pt/SSM

The SSM was carefully cleaned in ethanol, 20% HCl aqueous solution, and deionized water for 15 min with assistance of sonication to remove the impurities on the surface respectively. The MoS_2_/SSM was synthesized via a simple hydrothermal method. 0.19 g CH_4_N_2_S and 0.121 g Na_2_MoO_4_·2H_2_O were added to 30 mL of deionized water to form a homogeneous solution under vigorously stirring for 10 min. Then the cleaned SSM and solution were transferred into a 50 mL Teflon-lined stainless steel autoclave (Zhong Nuo Instrument Co., Ltd., Wuhan, China), sealed and maintained 220 °C for 24 h, then naturally cooling down to room temperature. Subsequently, the sample was taken out, rinsed in ethanol and deionized water respectively and dried at 50 °C for overnight. The MoS_2_/SSM was immersed in 20% H_2_SO_4_ solution for 10 min to pretreat the surface and then subjected to 200 CV cycles (150 mV s^−1^ between 0.4 and −1.0 V vs. SCE) using a graphite rod as a counter electrode to activate the electrode in the same electrolyte solution [13,29].

The Pt-MoS_2_/SSM was synthesized by a CV cycle method, which was carried out in a standard three-electrode cell containing 0.5 mol L^−1^ H_2_SO_4_ aqueous solution (pH = 0.03, 50 mL) at 20 °C using the activated MoS_2_/SSM, a Pt plate electrode, and a saturated calomel electrode (SCE) (Gaoss Union, Wuhan, China) as working, counter and reference electrodes, respectively. The CV experiment was performed with 200, 400, 600, 800 cycles between 0.6 and −1.2 V vs. SCE with a scan rate of 150 mV s^−1^. Subsequently, the electrodes were withdrawn from the solution, carefully rinsed with deionized water and dried at 50 °C. For comparative studies, Pt/SSM was synthesized via the same electrochemical method. The MoS_2_ and Pt loadings of Pt-MoS_2_/SSM were determined by inductively coupled plasma-optical emission spectrometry (ICP-OES, Optima 5300DV, Perkin Elmer, Richmond, California, CA, USA) to be ~0.14 mg cm^−2^ and ~0.13 mg cm^−2^, respectively. There is almost the same loading mass of MoS_2_ (0.14 mg cm^−2^) in MoS_2_/SSM and Pt (0.13 mg cm^−2^) in Pt/SSM electrode respectively according to the ICP-OES results.

### 2.3. Characterizations

The crystal phase analysis of the MoS_2_/SSM and Pt-MoS_2_/SSM electrocatalysts were performed by X-ray diffraction (XRD, XRD-7000S diffractometer, Shimadzu, Kyoto, Japan) using Cu K_α_ radiation at a scanning rate of 5° min^−1^ in the 2θ range from 10 to 80°. The morphology and structure of the samples were characterized using scanning electron microscopy (SEM, Hitachi S-4800 high-resolution scanning electron microscope, Kyoto, Japan) at an accelerating voltage of 15 kV and transmission electron microscopy (TEM, Hitachi H-7600 transmission electron microscope, Kyoto, Japan). The elemental composition of the Pt-MoS_2_ supported by SSM and the valence states of the metal elements were probed by an X-ray photoelectron spectroscopy (XPS, Thermo ESCALAB 250XI, Massachusetts, MA, USA). The Faradic efficiency of the electrocatalysts was determined by analyzing H_2_ produced at a current density of 50 mA cm^−2^. The gaseous products were detected using a GC-7920 Gas Chromatograph (Beijing Zhong Jiao Jin Yuan Technology Co., Ltd., Beijing, China) equipped with a TCD detector (N_2_ as carrier gas).

### 2.4. Electrochemical Measurements

All electrochemical measurements were conducted at room temperature with a CHI 660E electrochemical workstation (CH Instrument Co., Ltd., Shanghai, China) in a typical three-electrode system. The SSM loaded with MoS_2_, Pt, and Pt-MoS_2_ were directly used as working electrode, a graphite rod as counter electrode, and SCE as reference electrode. The electrolyte solutions were 0.5 mol L^−1^ H_2_SO_4_ aqueous solution and all the potentials reported in this paper were calibrated with the reversible hydrogen electrode (RHE) according to the equation: *E_RHE_* = *E_SCE_* + 0.2412 + 0.05916 × pH. All the polarization curves were measured at 5 mV s^−1^ by linear sweep voltammetry (LSV). The long-term stability tests were carried out by the chronopotentiometry in same conditions. There were IR compensations and no stirring in all measurements.

### 2.5. Calculation of TOFs for HER

The turnover frequency (*TOF*, s^−1^) for HER was calculated by following equation:

(1) TOF=j×A/(2×F×m)

where *j* is the current density (A cm^−2^) obtained at overpotential of 150 mV, *A* is the surface area of the electrode, *F* is the Faraday efficiency (96,485 C mol^−1^) and *m* is the number of catalyst active sites (in mol) based on the calculation in one CV cycle (from previous report [30]). The factor 1/2 is included considering that two electrons are required to form one hydrogen molecule from two protons.

## 3. Results

**Figure 1:**
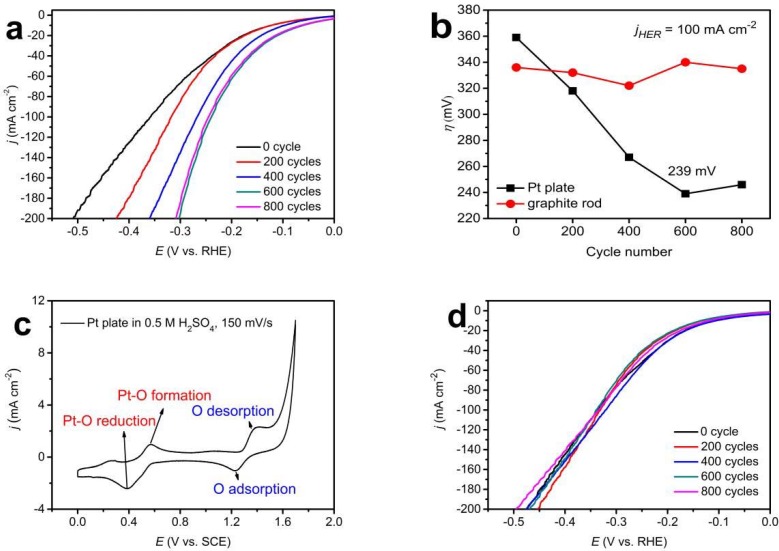
HER polarization curves of the MoS_2_/SSM after different numbers of CV cycles with (**a**) Pt plate counter electrode and (**d**) graphite rod counter electrode. (**b**) Dependence of the overpotentials of the samples on the cycle number with different counter electrode. (**c**) CV curve of the Pt plate obtained at a scan rate of 150 mV/s in 0.5 mol L^−1^ H_2_SO_4_ aqueous solution.

The HER polarization curves of Pt-MoS_2_/SSM fabricated by 200, 400, 600, 800 CV cycles with Pt as counter electrode were shown in Figure 1a. The polarization curve of MoS_2_/SSM (denoted as “0 cycle” in Figure 1a) is also included for comparison. The polarization curves changed remarkably when the CV cycles increased from 0 to 600 cycles. After 600 cycles, the catalytic performance became stable. The HER overpotentials at *j*_HER_ = 100 mA cm^−2^ as a function of cycle number used in the Pt-MoS_2_/SSM synthesis process is shown in Figure 1b. The enhancement of the HER activity should be attributed to the trace dissolved Pt species from the counter electrode and redeposition on the working electrode. To probe the formation process of Pt in MoS_2_/SSM, we used the same Pt plate as a working electrode to do the redox process in 0.5 mol L^−1^ H_2_SO_4_ aqueous solution with the scan rate of 150 mV s^−1^. As shown in Figure 1c, the representative peaks at 0.39 and 0.57 V vs. SCE should be ascribed to the formation/reduction of Pt oxide, respectively. Other peaks under larger potentials are rooted in electro adsorption/desorption of ‘O’ and oxygen evolution reaction (OER) according to the previous report [26]. By contrast, if a graphite rod was employed as the counter electrode during the Pt-MoS_2_/SSM synthesis process, the HER polarization curves remained stable even after 800 CV cycles, as shown in Figure 1d.

**Figure 2:**
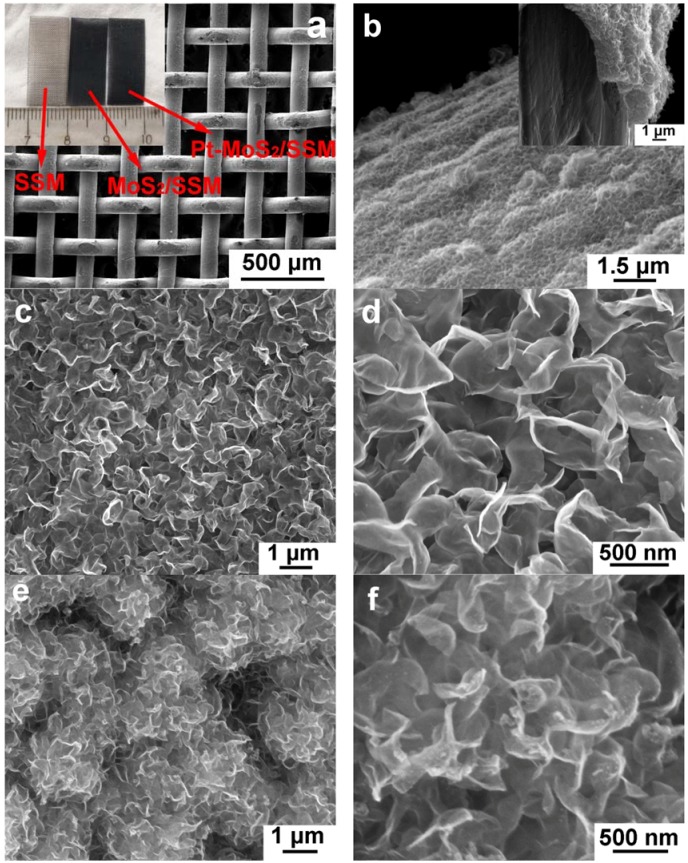
SEM images of the samples at top view (**a**) and side view (**b**) of Pt-MoS_2_/SSM. (**c**,**d**) High-resolution SEM images of MoS_2_/SSM composites. (**e**,**f**) Pt-MoS_2_/SSM composites. The upper inset in (**a**) shows a photograph that compares the SSM substrate before and after the hydrothermal and the electrochemical treatment.

The morphology of the as prepared MoS_2_/SSM was investigated by SEM, as shown in Figure 2. It is clearly observed that the SSM structure integrity is mostly retained after hydrothermal and electrochemical treatment (Figure 2a). The SSM color changed from silvery white to dark grey after hydrothermal treatment, with almost no further change after CV process, as shown in inset Figure 2a. The side view of high magnifications of the SSM was displayed in Figure 2b, suggesting that the MoS_2_ nanoflakes was successfully deposited on SSM with the thickness of ~1.5 μm. Figure 2c,d showed the high resolution SEM images of MoS_2_/SSM with nanoflakes of size between 300 to 800 nm. Figure 2e,f showed the high resolution SEM images of Pt-MoS_2_/SSM had similar morphology as MoS_2_/SSM. In both MoS_2_/SSM and Pt-MoS_2_/SSM, interconnected nanoflake stack structures were observed, which can provide abundant electrolyte channels for short ion diffusion/exchange paths and greatly enhance the active surface area for electrochemical reactions. It should be noticed that under the current magnification, no Pt nanoparticles were observed in the SEM of Pt-MoS_2_/SSM samples. The existence of Pt nanoparticles will be proven by the TEM studies discussed later.

**Figure 3:**
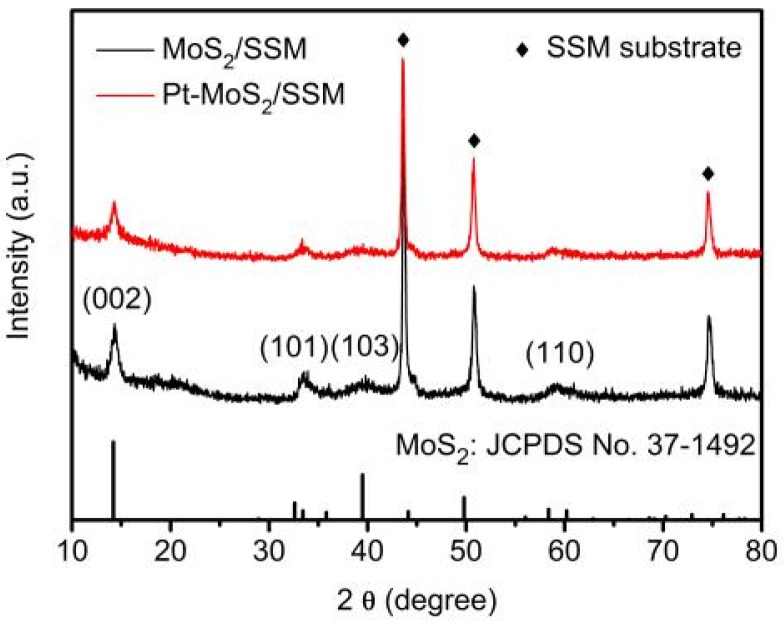
XRD pattern of the MoS_2_ nanoflakes (black) and Pt-MoS_2_ composites (red) supported by SSM substrate (rhombus).

Figure 3 shows XRD pattern of the MoS_2_ nanoflakes (black) and Pt-MoS_2_ composites (red) supported by SSM substrate (marked in rhombus). The diffraction peaks at 14.4°, 33.5°, 39.5° and 58.3° should be assigned to MoS_2_ (JCPDS No. 37-1492) after hydrothermal process [28]. The broadening of the (101), (103) and (110) peaks indicates that the lattice imperfections in the crystal structure. There are no peaks corresponding to metallic molybdenum or molybdenum oxides. Meanwhile, after the CV process, the characteristic peaks of Pt-MoS_2_ are observed with no obvious shift but a slight decrease in the intensity for the typical basal (002) planes of MoS_2_, implying the increase of interplanar spacing could happen after Pt nanoparticles insertion or CV process [20,29,31,32]. However, no signal of Pt is observed, which is attributed to the low mass ratio of Pt in the nanocomposite.

**Figure 4:**
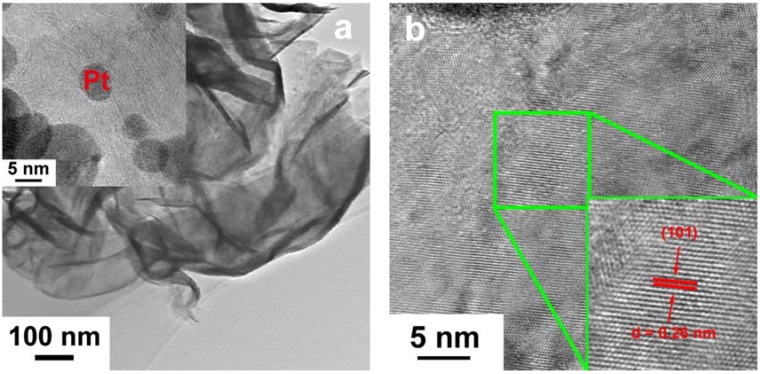

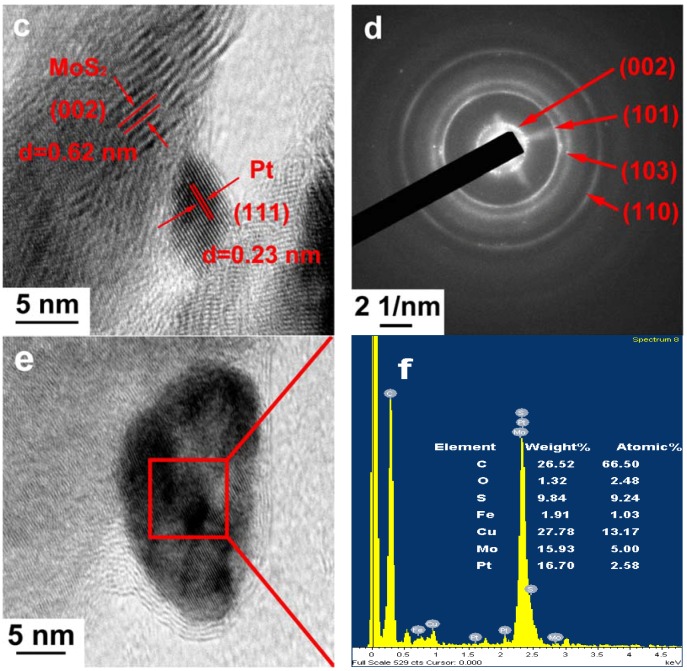
Low (**a**) and high (**b**,**c**,**e**) TEM images of Pt-MoS_2_ nanoflakes scratched off from SSM (Pt nanoparticles display in deep color) and corresponding SAED pattern (**d**); EDX spectrum (**f**) of Pt-MoS_2_ based on image (**e**).

In order to further elucidate the structure of the as prepared electrode, TEM and EDX (Energy Dispersive X-ray Spectroscopy) were conducted for Pt-MoS_2_ are shown in Figure 4. TEM image in Figure 4a shows the morphology of MoS_2_ layer, confirming that the thin nanoflakes structure of MoS_2_ with thickness of ~20 nm has been successfully synthesized. Obviously, Pt nanoparticles with a relatively deep color are distributed in the MoS_2_ layer with diameters between 3 nm and 15 nm, as shown in Figure 4. The high-resolution TEM images of Pt-MoS_2_ are illustrated in Figure 4b,c. The interplanar spacing of 0.62 nm and 0.26 nm should be assigned to the typical (002) and (101) lattice plane of MoS_2_, respectively. Moreover, the adjacent lattices spacing is 0.23 nm in Figure 4c, which is well in agreement with the (111) plane of Pt. As displayed in Figure 4d, the selected-area diffraction (SAED) pattern for Pt-MoS_2_ further indicates that the diffraction ring is well indexed to MoS_2_. Figure 4e shows the boundary details between Pt nanoparticle and MoS_2_. Combining with the TEM-EDX image of Pt nanoparticle showed in Figure 4f, all the results suggest that the successfully insertion of Pt species into MoS_2_ stacked structures.

**Figure 5:**
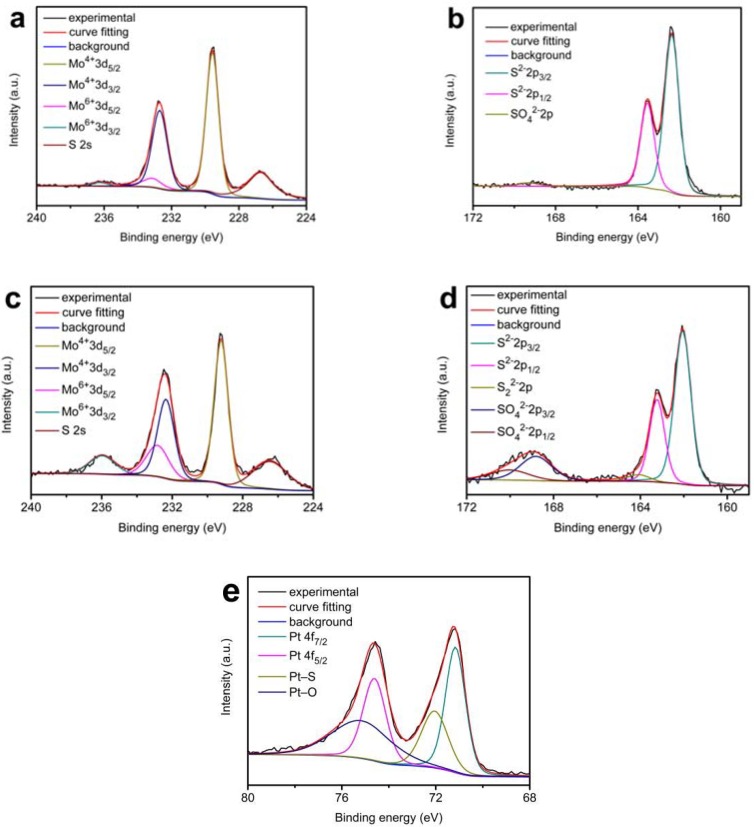
XPS spectra of MoS_2_/SSM (**a**,**b**) and Pt-MoS_2_/SSM (**c**–**e**): (**a**,**c**) Mo 3d, (**b**,**d**) S 2p, and (**e**) Pt 4f.

XPS experiments were carried out to further determine the chemical state of MoS_2_/SSM and Pt-MoS_2_/SSM catalysts. Figure 5a,b display the XPS spectra of Mo are 3d and S 2p of the MoS_2_/SSM. In the molybdenum spectrum of MoS_2_/SSM, two peaks are observed at approximately 229.6 eV and 232.7 eV, which can be attributed to typical Mo 3d_5/2_ and Mo 3d_3/2_ binding energies of Mo^4+^ oxidation state in all the materials respectively [15,20,29,32]. The peaks at 226.7 eV are indexed to S 2s [16]. Note that the presence of a shoulder in the Mo 3d_3/2_ peak indicates the Mo^4+^ ions are mixed with a few Mo^6+^ ions, which should be attributed to the surface oxidation of MoS_2_ in the air [15,32],. Meanwhile, the corresponding S 2p peaks were deconvoluted by fitting the data with the S 2p_3/2_ and S 2p_1/2_ at 162.4 eV and 163.5 eV suitably in Figure 5b. An extremely weak peak of the SO_4_^2−^ observed at 169.3 eV is due to the oxidation in the air. 

Compared with the MoS_2_/SSM, significant changes in both Mo and S spectra are observed owing to the insertion of platinum for Pt-MoS_2_/SSM. There are four peaks in the Mo 3d spectrum of Pt-MoS_2_/SSM in Figure 5c. Except the S 2p peaks at 226.4 eV, other peaks are ascribed to an overlap of two doublets of Mo^4+^ ions and Mo^6+^ ions 3d peaks. The ratio of Mo^6+^ to Mo^4+^ for Pt-MoS_2_/SSM is evaluated to be ~0.55 by taking a ratio of the intensities of the deconvoluted peaks. As compared to the ratio of Mo^6+^ to Mo^4+^ of ~0.10 for MoS_2_/SSM, it indicates that the structure of MoS_2_ has been changed and there are some platinum atoms incorporated into the MoS_2_ catalyst. The presence of a shoulder in the S 2p_1/2_ peak in Figure 5d reveals presence of a small amount of S_2_^2−^, which is a major active edge site of MoS_2_ [29,33,34,35,36]. Furthermore, the peak of SO_4_^2−^ markedly increases with the insertion of platinum, which likely be attributed to the accumulation of sulfur oxidation during CV process, agreeing with those in previous reports [32,37,38]. From the high resolution Pt 4f XPS peaks shown in Figure 5e, characteristic peaks of Pt 4f_7/2_, Pt 4f_5/2_ at 71.2 eV and 74.6 eV, respectively, should be attributed to metallic Pt nanoparticles. The Pt 4f peak shows two shoulders on the high binding energy side of the Pt 4f_5/2_ and Pt 4f_7/2_ peaks at a binding energy of 75.2 eV and 72.1 eV, which can be assigned to the Pt atoms linked to the oxygen and sulfur atoms respectively [39,40]. The formation of Pt–S bond can cause sulfur vacancies, and then enhance the catalytic activity of MoS_2_ inner planes [29,41,42].

**Figure 6:**
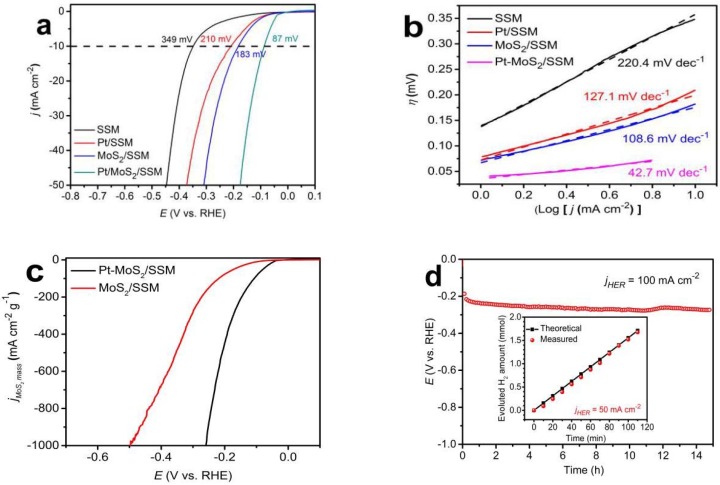
(**a**) HER polarization and corresponding (**b**) Tafel plots of Pt-MoS_2_/SSM, MoS_2_/SSM, Pt/SSM, and SSM electrodes in 0.5 mol L^−1^ H_2_SO_4_ aqueous solution at a scan rate of 5 mV s^−1^. (**c**) MoS_2_ mass activities plot and (**d**) durability test of Pt-MoS_2_/SSM electrode. Insert in (**d**) shows the amount of theoretically calculated and experimentally measured H_2_ as a function of time for Pt-MoS_2_/SSM at the constant current densities of 50 mA cm^−^^2^.

The HER catalytic activities of the Pt-MoS_2_/SSM electrode have been further characterized with a three electrode electrochemical system in 0.5 mol L^−1^ H_2_SO_4_ aqueous solution by linear sweep voltammetry (LSV). Figure 6a shows the polarization curve of the as-obtained composite electrodes at a scan rate of 5 mV s^−1^. The as-prepared electrodes of MoS_2_/SSM and Pt/SSM exhibit overpotentials of 183 mV and 210 mV, respectively at a current density of 10 mA cm^−2^. The Pt-MoS_2_/SSM shows much improved HER activity of overpotentials of 87 mV to achieve the same current density. Moreover, the overpotential values are 515 mV for SSM, 434 mV for Pt/SSM, 379 mV for MoS_2_/SSM, and 225 mV for Pt-MoS_2_/SSM, at the current density of 100 mA cm^−2^. The corresponding Tafel plots of the four electrodes are displayed in Figure 6b. As expected, the Tafel slope of Pt-MoS_2_/SSM electrode is only 42.7 mV dec^−1^, which is much lower than those of the MoS_2_/SSM (108.6 mV dec^−1^), Pt/SSM (127.1 mV dec^−1^), and SSM (220.4 mV dec^−1^) electrodes, suggesting fast kinetics of the Pt-MoS_2_/SSM electrode. In addition, the turnover frequency value of the Pt-MoS_2_/SSM was calculated to be 0.27 s^−1^ at an overpotential of 150 mV, which is higher than that of the MoS_2_/SSM (0.08 s^−1^) and Pt/SSM (0.05 s^−1^). The mass-specific activities of MoS_2_ (*j_mass_*) in MoS_2_/SSM and Pt-MoS_2_/SSM samples can be calculated by the following equation [25]:

(2) $$j_{mass}^{MoS_{2}/SSM}=\frac{j_{area}^{MoS_{2}/SSM}-j_{area}^{SSM}(mA/cm^{2})}{mass_{MoS_{2}}(mg/cm^{2})}$$

(3) $$j_{mass}^{Pt-MoS_{2}/SSM}=\frac{j_{area}^{Pt-MoS_{2}/SSM}-j_{area}^{Pt/SSM}(mA/cm^{2})}{mass_{MoS_{2}}(mg/cm^{2})}$$

Based on the equation, the *j_mass_* at different potentials was be calculated. As shown in the Figure 6c, the *j_mass_* of Pt-MoS_2_ is higher than the value of MoS_2_ for all potentials, indicating the MoS_2_ mass activities were increased after Pt was introduced. According to these results, it is reasonable to believe that the increased activity of HER should be attributed to the change of the MoS_2_ structure with insertion of platinum and a strong synergistic effect between Pt nanoparticles and the MoS_2_ nanoflakes.

The durability of the Pt-MoS_2_/SSM electrode is evaluated in long-term bulk electrolysis via chronoamperometry. As seen from Figure 6d, the catalytic electrode shows almost a horizontal curve at a current density of 100 mA cm^−2^ in 0.5 M H_2_SO_4_ aqueous solution. There is only a small increasement in potential of 32 mV at the current density of 100 mA cm^−2^ for 15 h, suggesting the high stability of Pt-MoS_2_/SSM electrode. The insert image in Figure 6d shows the theoretically calculated gas amount equals well to the experimentally measured values, suggesting that the Faradic efficiency of Pt-MoS_2_ electrocatalyst was about 100%. 

## 4. Conclusions

In conclusion, using a simple CV cycling process, we have successfully synthesized Pt-MoS_2_/SSM catalyst, which comprises of Pt nanoparticles inserted into the stacked structures of MoS_2_ supported by SSM. The binder-free Pt-MoS_2_/SSM electrode can be directly used as a catalytic electrode for HER. Compared to SSM, Pt/SSM and MoS_2_/SSM, Pt-MoS_2_/SSM exhibits much higher electrocatalytic activity for HER. It exhibits a very low over potentials of 87 and 225 mV for current density of 10 and 100 mA cm^−2^, respectively, and a good durability in acid. The MoS_2_ in Pt-MoS_2_/SSM also has higher mass-specific catalytic activity than that in MoS_2_/SSM. The superior performances of Pt-MoS_2_/SSM are mainly ascribed to its unique nanostructure and the synergistic effect between the nanoparticles and the MoS_2_ nanoflakes. This work provides a promising approach for synthesizing advanced catalysts for energy conversion and storage applications.
